# Application and value of anxiety and depression scale in patients with functional dyspepsia

**DOI:** 10.1186/s40359-024-01744-3

**Published:** 2024-04-30

**Authors:** Yejiao Ruan, Hao Lin, Xinru Lu, Yiying Lin, Jian Sun, Cengqi Xu, Lingjun Zhou, Zhenzhai Cai, Xiaoyan Chen

**Affiliations:** 1 The Second Affiliated Hospital & Yuying Children’s Hospital of Wenzhou Medical University, 1111 East Wenzhou Dadao, Longwan District, Wenzhou, 325000 Zhejiang China; 2 The Second Clinical Medical College, Wenzhou Medical University, Wenzhou, Zhejiang China

**Keywords:** Anxiety symptoms, Depression symptoms, Assessment, Functional disease, Functional dyspepsia

## Abstract

**Background:**

Patients with functional dyspepsia (FD) cannot be assessed for their mental health using a suitable and practical measure. The purpose of the study is to investigate the usefulness of several anxiety and depression scales in patients with FD, offering recommendations for clinical identification and therapy.

**Methods:**

From September 2021 to September 2022, patients were sought and selected. The psychological symptoms were assessed using ten depression or anxiety questionnaires. The receiver operating characteristic (ROC) curve, Spearman analysis, Pearson correlation analysis, and single factor analysis were applied.

**Results:**

Prospective analysis was performed on 142 healthy individuals and 113 patients with FD. In the case group, anxiety and depression symptoms were more common than in the control group, and the 10 scales showed strong validity and reliability. HAMD had the strongest connection with the PHQ-9 score on the depression scale (0.83). The score correlation between SAS and HAMA on the anxiety analysis scale was the greatest at 0.77. The PHQ-9, SAS, HAMD, and HAMA measures performed exceptionally well in detecting FD with anxiety or depression symptoms (AUC = 0.72, 0.70, 0.70, 0.77, and 0.77, respectively).

**Conclusions:**

PHQ-9, SAS, HAMD, and HAMA scales have good application performance in FD patients. They can assist gastroenterologists in evaluating anxiety and depression symptoms, and provide reference and guidance for subsequent treatment.

## Background

One of the most common functional gastrointestinal disorders is functional dyspepsia (FD) [1, 2]. According to ROME criteria, population-based studies reported that the overall global pooled prevalence of FD in adults from 1990 to 2022 was 8.4%, which was even higher in developing countries (9.0%) [3]. Low cure rates, frequent flare-ups, and persistent symptoms are all characteristics of FD that impair patients’ quality of life and cause a greater financial healthcare burden which reaches $1.84 billion per year in the USA [4–6]. Existing studies have demonstrated that FD is strongly correlated with psychological and mental factors [7, 8]. However, there are currently few ways to identify FD patients who have psychiatric issues, and psychological assessments can offer a scientifically valid basis for detection [9]. More than 10 different psychological assessment scale subtypes are currently employed in clinical assessments of psychological disorders, none of which are determined as the golden standards in clinical practice yet due to the lack of systematic research [10]. Investigating useful psychological assessments for the early detection of anxiety and depression symptoms in gastrointestinal patients is crucial. This study examined and compared the effectiveness of various psychological measures used with FD patients to offer a clinical foundation for the selection of suitable evaluation instruments for the clinical practice of doctors. It may help to further enhance the quick recognition, diagnosis, and treatment of FD by medical doctors as well as enhance the clinical prognosis of FD patients.

## Materials and methods

### Study population

From September 2022 to March 2023, the researchers prospectively gathered pertinent data from outpatients and healthy individuals receiving physical examinations at the Second Affiliated Hospital of Wenzhou Medical University. Rome IV criteria were used to diagnose patients with functional dyspepsia, and the healthy individuals who completed physical tests in the hospital during the same period made up the control group.

### Inclusion criteria

Inclusion criteria were: aged 18–80; originating in the gastroduodenal region, except for organic, systemic, or metabolic diseases that could explain the symptoms, containing at least 1 of the following 4 symptoms (upper abdominal pain, epigastric burning sensation, postprandial fullness, and early satiety), with a disease duration of more than 6 months, and symptoms have occurred in the past 3 months. In all cases, endoscopy was performed to exclude organic disease.

### Exclusion criteria

Exclusion criteria were: pregnant or lactating; history of any other diagnosed gastrointestinal disorders which can explain dyspeptic symptoms, such as esophagitis, gastritis, ulcer disease, etc.; history of severe major abdominal surgery; history of psychotic disorders or other severe psychiatric disorders; history of other severe organic disorders, such as pancreaticobiliary disease, metabolic disease or liver dysfunction; currently using drugs with proven gastrointestinal adverse effects, such as steroids or nonsteroidal anti-inflammatory drugs; or declined to participate in this study.

All study participants gave a written informed agreement allowing their data to be recorded and utilized in the study, and the study was carried out with the Ethics Committee of the Second Affiliated Hospital of Wenzhou Medical University’s clearance. The study adhered to the confidentiality of patient information concept, and the completed questionnaire was immediately collected.

### Related scales

Hamilton Anxiety Rating Scale (HAMA): Compiled by Hamilton in 1959, it includes 14 items, all of which are rated on a 5-point scale from 0 to 4 [11].

Hamilton Depression Rating Scale (HAMD): Developed by Hamilton in 1960, it is divided into three versions. A 24-item version was used in this study [12].

Self-Rating Anxiety Scale (SAS): Prepared by William WK Zung, contains 20 items, divided into 4 grades [13].

Self-Rating Depression Scale (SDS): It is a self-rating scale with 20 items and divided into 4 grades. The prototype is the depression scale compiled by WKZung (1965) [14].

Patient Health Questionnaire 9(PHQ-9): It is a 9-item scale based on the DSM-IV diagnostic criteria, using a 4-point scoring method of 0 to 3 points [15].

The 7-item Generalized Anxiety Disorder Scale (GAD-7): Developed by Spizer et al. (2006), there are 7 items in total, and it is a 4-point rating on a scale of 0–3 [16].

The 90s four-question questionnaire of depression (D90-4): The subjects were asked to answer four questions within the 90s, and depression could be quickly screened according to the answers.

The 90s four-question questionnaire for anxiety (A90-4): The subjects were asked to answer four questions within the 90s, and anxiety could be quickly screened according to the answers.

General Hospital Anxiety and Depression Scale (HADS): The HADS consists of 14 items to calculate the patient’s composite score, seven of which are anxiety-related (HADS-D) and the other seven are depression-related (HADS-A) [17].

### Data collection

Following completion of the baseline personal information questionnaire, participants were given the SDS, HAMD, HADS-D, PHQ-9, and D90-4 depression measures as well as the SAS, HAMA, HADS-A, GAD-7, and A90-4 anxiety scales. The patients voluntarily filled out the self-rated scales after being explained by experienced researchers. Individually, both subjects and professionally trained researchers evaluated the clinician-rated scales. For the same patient, all scales were required to be completed in one day and there shouldn’t be more than a 30-minute gap between the filling times of two scales to prevent cross-talk between them and patient boredom from having too many scales.

### Statistic analysis

All statistical analyses were performed by SPSS 22.0. Continuous variables with normal distribution are expressed as mean standard deviation, and those with an abnormal distribution are expressed as median (upper and lower quartiles). Frequencies and percentages are used to represent categorical variables. Comparisons between groups were performed using the χ2 test and *t* test. Both Spearman and Pearson correlation analysis were used to perform the correlation analysis. GraphPad software was used to construct the receiver operating characteristic (ROC) curves for each of the 10 scales. The threshold for statistical significance was set at *p* < 0.05.

## Results

### Patient characteristics

This study examined 305 instances in total, and it only included the 255 individuals who fully completed the ten-item measure. The average age was 39.5 ± 11.1, and there were 114 females and 141 males. The case group consisted of 142 people with an average age of 38.2 ± 11.4 years, while the normal control group had 142 people with an average age of 41.1 ± 10.5 years. While there was no statistical difference in the gender ratio between the two groups (*p* > 0.05), the proportion of senior-year rent samples in the control group was higher than that in the case group (age > 40 years). Details can be found in Table 1.

**Table 1:** Comparison of general conditions of the two groups of patients

Group		FD(%)	Control(%)	*p*
Gender	Male	75(52.8)	66(58.4)	0.372
	Female	67(47.2)	47(41.6)	
Age	≤ 40	87(61.3)	55(48.7)	0.044
	>40	55(38.7)	58(51.3)	

### Reliability and validity tests

Overall, the validity and reliability of all five anxiety and depression scores were adequate. With the exception of D90-4 and A90-4, all scales demonstrated adequate internal consistency, with Cronbach’s alpha values for the four depression scales ranging from 0.71 to 0.87 and the four anxiety scales from 0.70 to 0.90. Except for the D90-4 and A90-4, the scales fared satisfactorily in terms of validity. The four depression scales had Spearman-Brown values greater than 0.80, whereas the four anxiety scales had values greater than 0.85. In general, the scales D90-4 reliability (0.68), validity (0.65), and A90-4 reliability (0.68) performed well. Details are provided in Tables 2 and 3.

**Table 2:** Reliability and validity analysis of five depression scales

	SDS	HAMD	HADS-D	PHQ-9	D90-4
Cronbach’s A validity	0.761	0.848	0.766	0.864	0.586
Spearman-Brown reliability	0.880	0.851	0.817	0.887	0.656

**Table 3:** Reliability and validity analysis of five anxiety scales

	SAS	HAMA	HADS-A	GAD-7	A90-4
Cronbach’s A validity	0.705	0.890	0.815	0.904	0.647
Spearman-Brown reliability	0.851	0.911	0.861	0.890	0.677

### Analysis of depression levels in FD patients

142 FD patients were assessed using the HAMD scale, and depression symptoms were shown to account for 41.5% (59/142), with severe depression symptoms (HAMD score > 24) accounting for 11.3% (16/142). In the control group, the percentage of depressed patients was 9.7%(11/113) and 2.7%(3/113) of patients with severe depression symptoms. The differences in the prevalence of depression symptoms between the two groups were statistically significant (*p* < 0.05). Besides, 61.3%, 36.6%, and 56.3% of FD patients were detected with depression symptoms by the SDS, HADS-D, and PHQ-9 self-rated scales, which were significantly higher than those in the control group, and the differences were statistically significant (*p* < 0.05), as shown in Table 4.

**Table 4:** Analysis of differences in demographic characteristics and depression scale scores

	SDS	*p*	HAMD	*p*	HADS-D	*p*	PHQ-9	*p*	D90-4	*p*
Gender		0.131		2.659		0.225		0.033		0.105
Male (*n* = 141)	49.8 ± 0.88		9.44 ± 0.61		5.94 ± 0.40		4.28 ± 0.38		1.02 ± 0.92	
Femal (*n* = 114)	51.98 ± 1.17		12.23 ± 0.89		5.30 ± 0.40		5.63 ± 0.52		1.25 ± 0.11	
Age		0.352		2.472		0.633		0.013		0.093
≤40 (*n* = 142)	51.37 ± 11.64		11.84 ± 8.81		5.54 ± 4.21		5.58 ± 5.53		1.23 ± 1.20	
>40 (*n* = 113)	50.03 ± 11.21		9.24 ± 7.71		5.80 ± 4.24		4.01 ± 4.50		0.99 ± 1.05	
Group		0.000		5.473		0.120		0.000		0.000
Control (*n* = 113)	47.95 ± 1.00		7.62 ± 0.64		5.19 ± 0.39		2.95 ± 0.35		0.82 ± 0.10	
FD (*n* = 142)	53.03 ± 0.98		13.13 ± 0.74		6.02 ± 0.36		6.43 ± 0.45		1.37 ± 0.10	

**Fig. 1:**
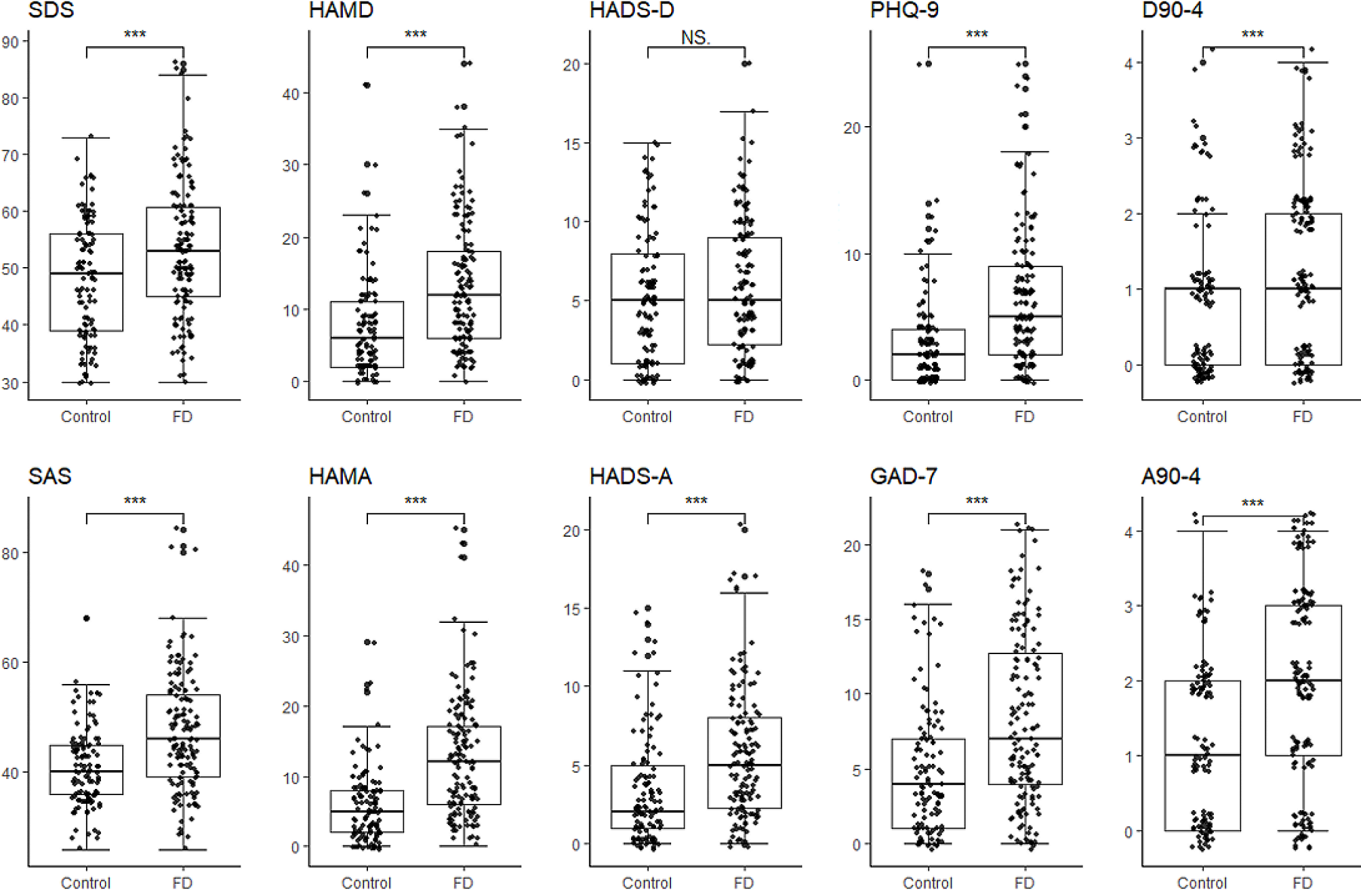
Comparison of the scores of the two groups in the ten scales. *, <0.05; **, <0.01; ***, <0.001

In addition to displaying a greater prevalence, FD patients also had higher levels of depression. With a mean score of 47.95 ± 1.00 on the SDS, 7.62 ± 0.64 on the HAMD, 2.95 ± 0.35 on the PHQ-9, and 0.82 ± 0.10 on the D90-4, FD patients considerably outperformed healthy controls on most depression scales, as shown in Fig. 1; Table 4. There were significant differences in HAMD and PHQ-9 scores between groups based on age and gender (*p* < 0.05). In comparison to the younger age group, the older age group reported greater HAMD and PHQ-9 scores. The PHQ-9 and HAMD scores of the female group were higher than those of the male group.

### Analysis of anxiety levels in FD patients

The five scales supported the fact that FD patients had higher levels of anxiety than the general population. The average SAS in the FD group score is 47.13 ± 0.88, HAMA is 5.72 ± 0.48, and GAD-7 is 4.84 ± 4.84. The distinction from the control group was statistically significant (*p* < 0.05). More information is available in Table 5.

**Table 5:** Analysis of differences in demographic characteristics and anxiety scale scores

	SAS	*p*	HAMA	*p*	HADS-A	*p*	GAD-7	*p*	A90-4	*p*
Gender		0.373		0.012		0.126		0.028		0.003
Male	43.87 ± 0.73		8.45 ± 0.56		4.26 ± 0.30		6.05 ± 0.44		1.43 ± 0.11	
Femal	44.96 ± 1.02		10.96 ± 0.86		5.02 ± 0.40		7.56 ± 0.53		1.92 ± 0.12	
Age, years		0.598		0.032		0.260		0.065		0.001
≤40	44.65 ± 10.59		10.52 ± 8.03		4.85 ± 3.87		7.29 ± 5.09		1.89 ± 1.23	
>40	44.00 ± 8.50		8.38 ± 7.72		4.28 ± 4.04		6.02 ± 5.86		1.35 ± 1.37	
Group		0.000		0.000		0.000		0.000		0.000
Control	40.88 ± 0.68		5.72 ± 0.48		3.36 ± 0.32		4.84 ± 0.41		1.22 ± 0.10	
FD	47.13 ± 0.88		12.64 ± 0.71		5.58 ± 0.34		8.23 ± 0.48		1.99 ± 0.12	

There were statistically significant variations in HAMA, GAD-7, and A90-4 scores between groups, as seen in Fig. 1, according to age and gender. The elder group had higher HAMA, GAD-7, and A90-4 scores, while women had higher HAMA, GAD-7, and A90-4 scores than men. Statistics showed that the difference was significant (*p* < 0.05). In contrast, there was no scientifically significant variation in the SAS and HADS-A scores across age and gender groups (*p* > 0.05).

### Comparative analysis of different scales

**Fig. 2:**
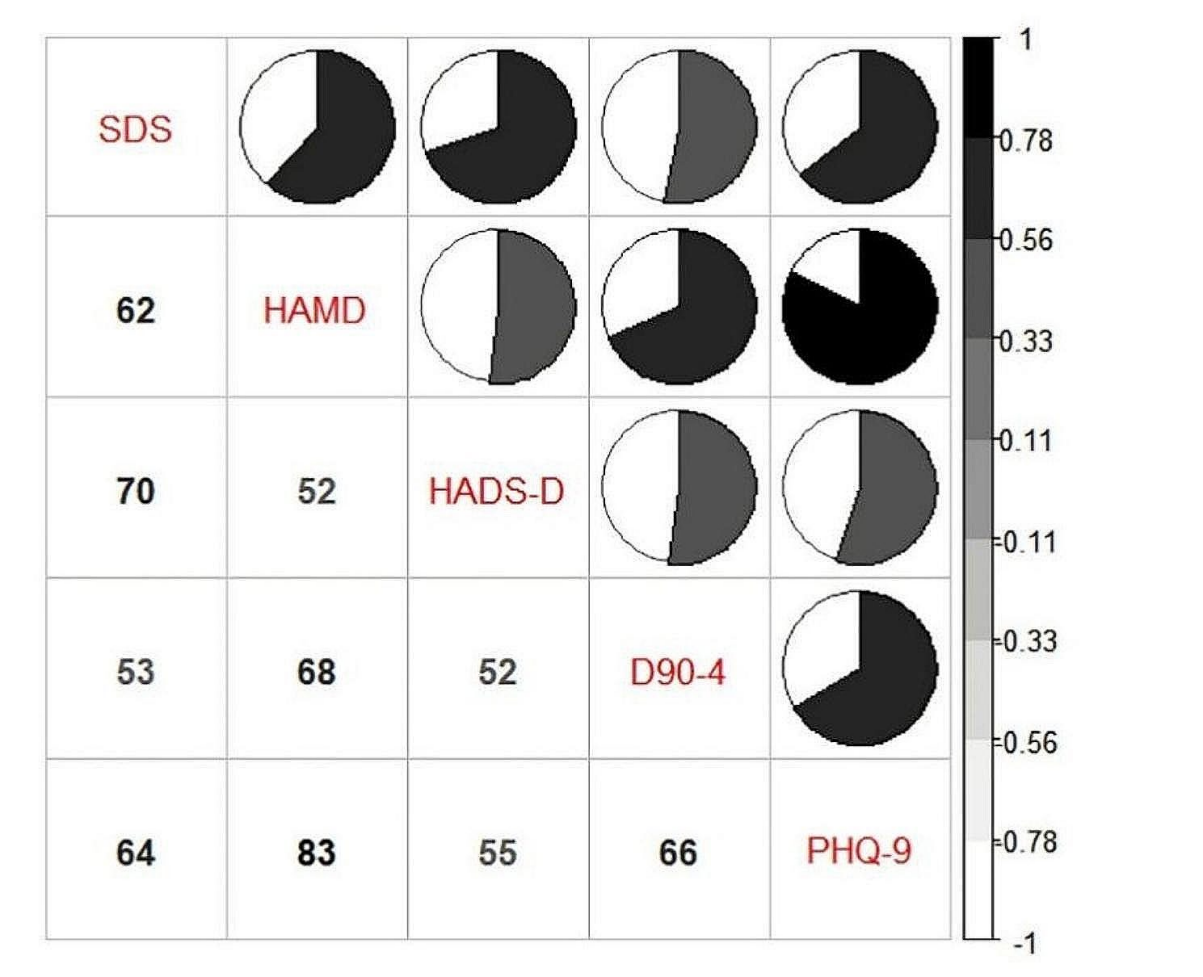
Correlation analysis of scores among five depression scales. Numbers represented the correlation(%). The correlation was reflected by the area and color depth of the pie chart

**Fig. 3:**
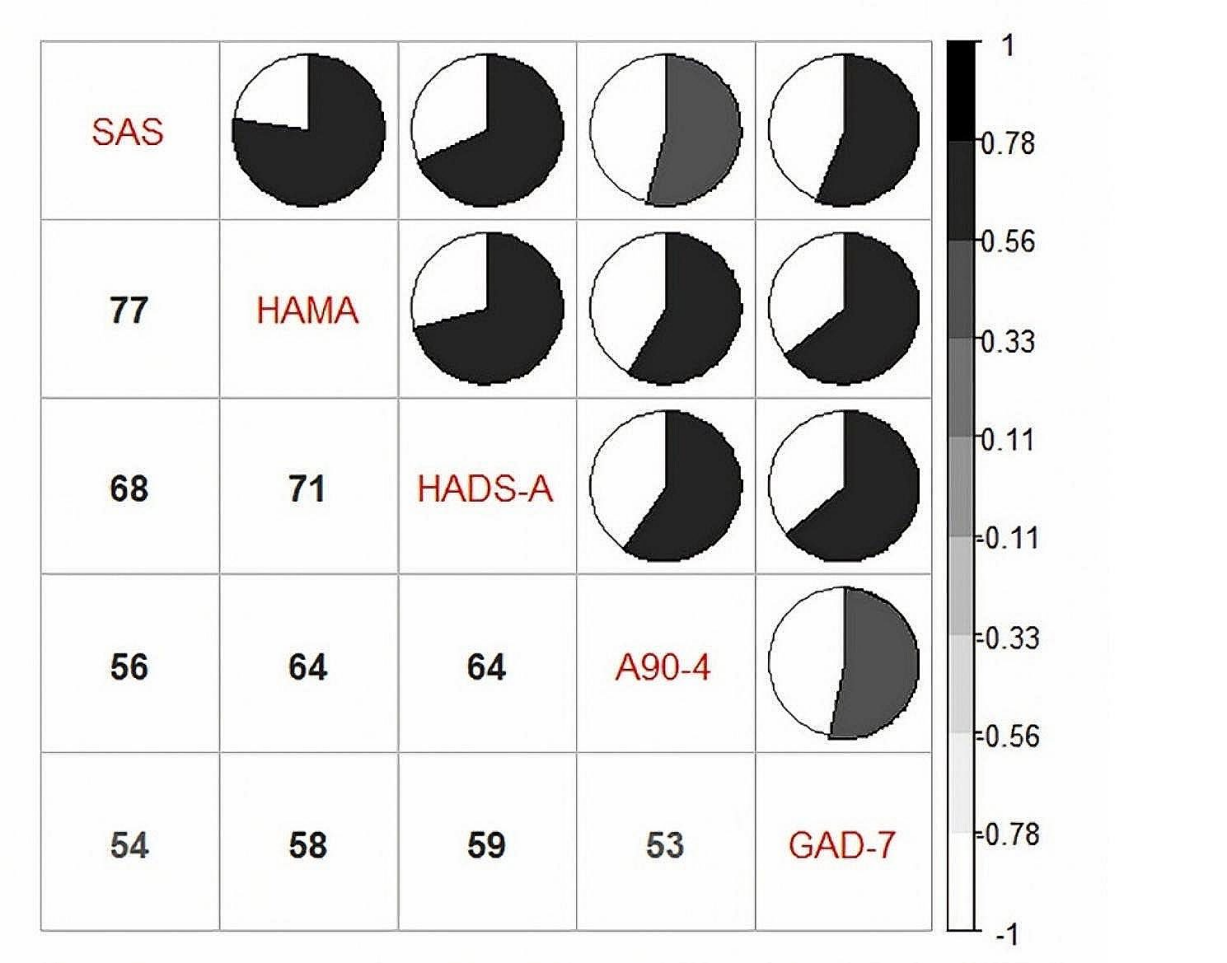
Correlation analysis of scores among five anxiety scales. Numbers represented the correlation(%). The correlation was reflected by the area and color depth of the pie chart

The dependability of the data was verified by the correlation index for all pairwise comparisons being greater than 0.5, as shown in Fig. 2, which compared the scores of the five depression scales for correlation. PHQ-9 demonstrated the strongest association with the HAMD score of all of them, at 0.83. The score correlations between the five scales in the assessment of anxiety measures, as shown in Fig. 3, ranged from 0.53 to 0.77, with the best score correlation occurring between SAS and HAMA and the relatively worst correlation being between A90-4 and GAD-7.

### Assess patients with clinical FD by anxiety and depression scales

**Fig. 4:**
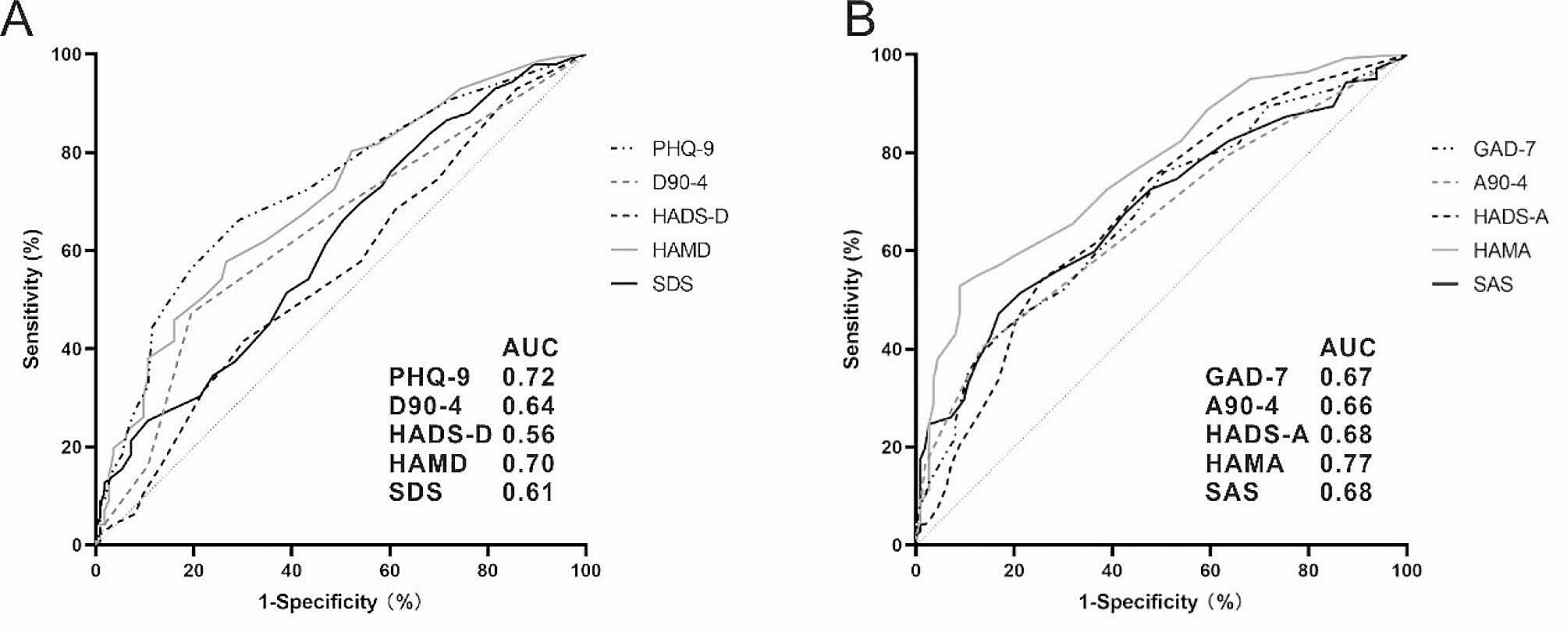
ROC curves for the correlation analysis between the scales and the diagnosis of FD. (**A**) ROC curve for the depression scale; (**B**) ROC curve for the anxiety scale

As shown in Fig. 4, with the application of ROC curve analysis, the SDS, HAMD, HAMD, and SDS scales’ AUCs ranged from 0.56 to 0.72 (*p* < 0.05), while the SAS, HAMA, HADS-A, A904, and GAD-7 scales’ AUCs ranged from 0.67 to 0.77. Generally, the anxiety scales demonstrated a better capability to detect FD. The three best-performing scales were the PHQ-9 (AUC = 0.72, *p* < 0.05), HAMD (AUC = 0.70, *p* < 0.05), and HAMA (AUC = 0.77, *p* < 0.05).

## Discussion

Recently, the significant role of psychosocial factors in functional dyspepsia has been acknowledged by mainstream researchers [18]. A significant association between anxiety, depression, and functional dyspepsia was also demonstrated by a meta-analysis from 2023 [19]. In our study, depression symptoms were shown to account for 41.5% of HAMD, 61.3% of SDS, 36.6% of HADS-D, and 56.3% of PHQ-9. The scores of anxiety symptoms were shown to account for 47.13 ± 0.88 by SAS, 5.72 ± 0.48 by HAMA, and 4.84 ± 4.84 by GAD-7. No matter the scale employed, the proportion of anxiety or depression symptoms in FD patients was considerably larger than in control groups, which is essentially in line with previous research. Additionally, FD patients experienced considerably more severe anxiety symptoms and despair than healthy controls. Our findings highlight the fact that individuals with FD experience more severe cases of anxiety and depression symptoms than people without FD, and they also point to a substantial association between FD and anxiety and depression symptoms. There is growing attention in how psychosocial variables affect the development and progress of FD. Functional gastrointestinal discomfort may be a somatic symptom of psychosomatic illnesses, which exacerbates the patients’ psychosomatic illnesses [10, 20]. Bidirectional brain-gut communication is one of the proposed pathways [21]. These correlation investigations indicate that it might be beneficial to treat FD by quickly and accurately assessing the psychosomatic symptoms of FD patients [20, 22]. The prognosis and regression of FD patients are impacted by the lack of objective criteria for assessing the psychological symptoms of FD patients in clinical practice and the lack of expertise in identifying and treating psychological illnesses among the majority of gastrointestinal practitioners. The two scales that psychiatrists use most frequently to identify bipolar disorder are the HAMD and HAMA. These two scales must be administered by psychiatric psychologists[23]. They will become biased and lose their effectiveness when non-professionals use them, which has a negative impact on non-psychiatrist practitioners and is harmful to the management and treatment of the illness.

There are currently more than 10 psychological evaluation scales used in clinical assessments of psychiatric illnesses in addition to HAMD and HAMA. It is challenging for gastroenterologists to screen patients for psychological disorders and is detrimental to the management and treatment of diseases since there is no relevant research on the effectiveness of various psychological evaluation samples used in digestive-related diseases. We evaluated and compared the score correlations, assessment consistency, reliability, and validity of the remaining four anxiety measures (SAS, HADS-A, GAD-7, and A90-4) and four depression scales (SDS, HADS-D, HPQ-9, and D90-4) in this study, which uses the author’s HAMA and HAMD as the standard. All of the measures we examined had strong associations. Patients with mental health issues can be assessed by choosing one of them. PHQ-9 demonstrated the strongest connection with HAMD (rs = 0.83, *p* < 0.05) among all of the depression scores. The association between SAS and HAMA was the strongest among the anxiety scales (rs = 0.77, *p* < 0.05). The clinician-rated measures used in psychiatric diagnosis are more consistently measured by the two scales, PHQ-9 and SAS, which seem to be simple to complete. We propose that doctors could early screen for anxiety and depression symptoms in FD patients using these two scales in addition to HAMA and HAMD.

Although the pooled prevalence of FD gradually decreased due to increasing medical level and improved life quality from 1990 to 2020 (12.4% [8.2–18.3] in 1990–2002 versus 7.3% [6.1–8.7] in 2013–2020) [3], FD-related health care operations are still imposing a significant financial burden on the healthcare system, whose global annual cost exceeds $18 billion [24, 25]. After considering for something like the price of medical care, drug rehab, and time off work, the average annual cost per FD patient in the US is $699 [4]. Patients with FD who are depressed or anxious have more severe physical symptoms, require more medical care, and spend more time recovering from sickness [1]. This underlines how crucial it is to promptly recognize and detect FD patients who are depressed and anxious. By analyzing the ROC curves of the scales in FD patients and healthy groups, we discovered that all 10 scales under investigation showed a strong association among FD with depression and anxiety symptoms, with the PHQ-9 (AUC = 0.72), HAMD (AUC = 0.70), and HAMA (AUC = 0.77) scales being particularly capable of detecting FD. This suggests that detecting patients with anxiety and depression symptoms aids in FD patient diagnosis. The identification of FD in the population through the detection of patients with anxiety and depression symptoms is also useful, and the patient’s state is improved with the support of a gastroenterologist [26–28]. Consequently, there is to believe that early detection and treatment of such people will assist in halting the progression of the disease and preventing the disease from upgrading from a functional to an organic morbid symptom.

Numerous factors could have an impact on the original study outcomes. Due to the retrospective nature of this study, flaws like selection bias may be unavoidably introduced. Second, this study used a small number of participants and was done at a single center, both of which may have introduced some sample bias. To investigate these topics in greater depth, additional studies with numerous centers and sizable samples are required. Furthermore, it is still unclear what role mental aspects have in the pathophysiology and prognosis of FD patients, and more research is required to establish the causative link [29–31]. Perhaps the facts and recommendations from our study will help figure out the causes of FD and the way to manage it.

## Conclusion

Functional dyspepsia patients are more prone than healthy people to experience both combined anxiety and depression symptoms, and their anxiety and depression symptoms are more severe. Ten psychological scales were used, and results in FD patients showed good correlations. The PHQ-9, SAS, HAMD, and HAMA scales all performed well in spotting FD when it was present with anxiety or depression symptoms. In order to assess anxiety and depression symptoms in patients, doctors might use the SAS and PHQ-9 scales as a guide and point of reference.

## Data Availability

The datasets generated for this study are available on request to the corresponding author.
